# Supplementary material for: Supramolecular catalyst with [FeCl4] unit boosting photoelectrochemical seawater splitting via water nucleophilic attack pathway
Source: Nat Commun. 2024 Mar 6;15:2023. doi: 10.1038/s41467-024-46342-4 (PMC10918074; doi:10.1038/s41467-024-46342-4)
Supplement: Supplementary file 1 — Supplementary Information [file 41467_2024_46342_MOESM1_ESM.pdf]

Supplementary Information for

# Supramolecular Catalyst with $[FeCl_4]$ Unit Boosting Photoelectrochemical Seawater Splitting via Water Nucleophilic Attack Pathway

Miao et al

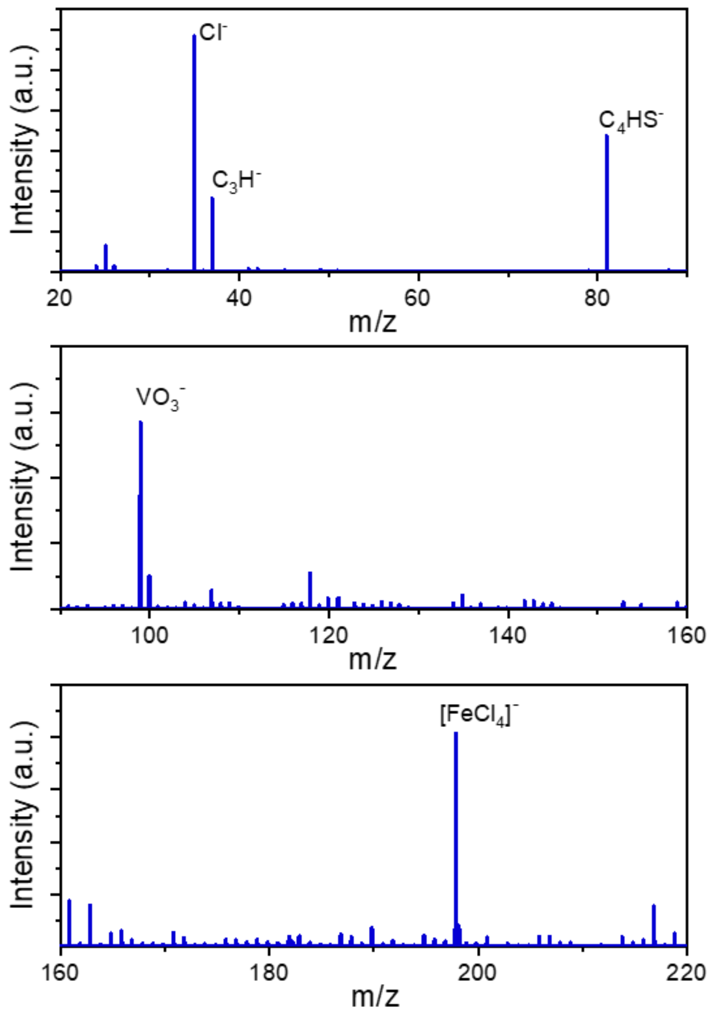


Supplementary Fig. 1. Negative TOF-SIMS spectra of the PTh/BVO.

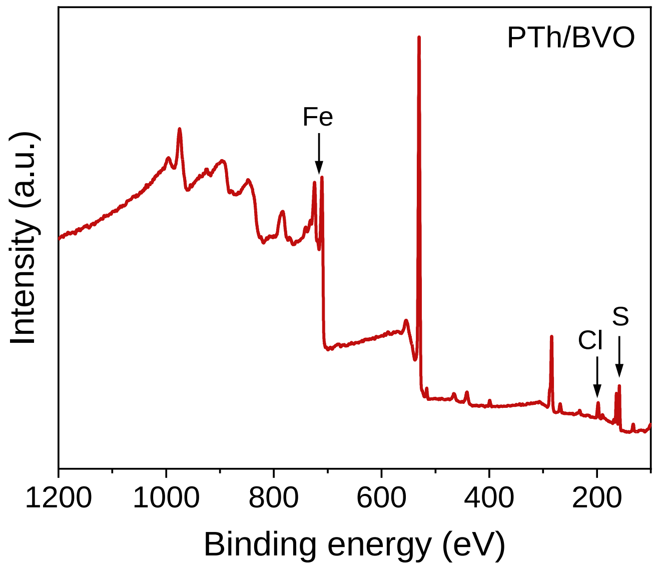


Supplementary Fig.2. XPS survey spectra of PTh/$BiVO_4$.

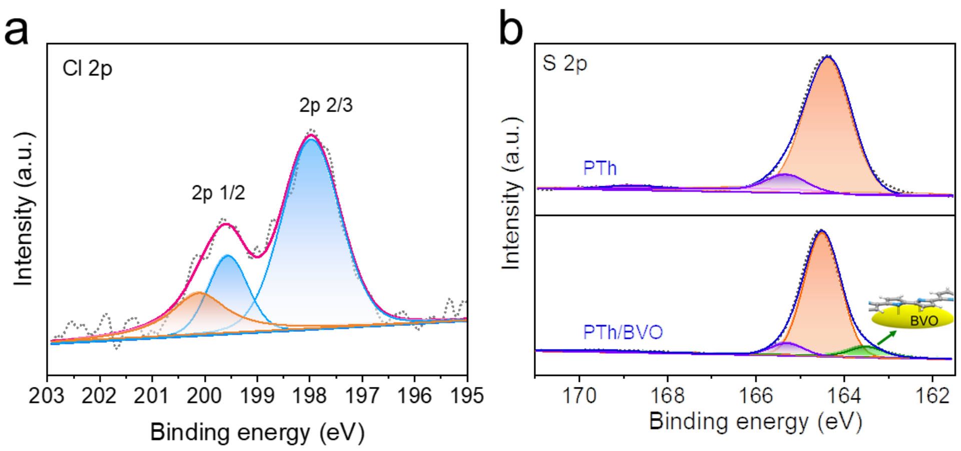


Supplementary Fig. 3. XPS spectra of PTh and PTh/BVO in selected energy areas: a) Cl 2p, b) S 2p.

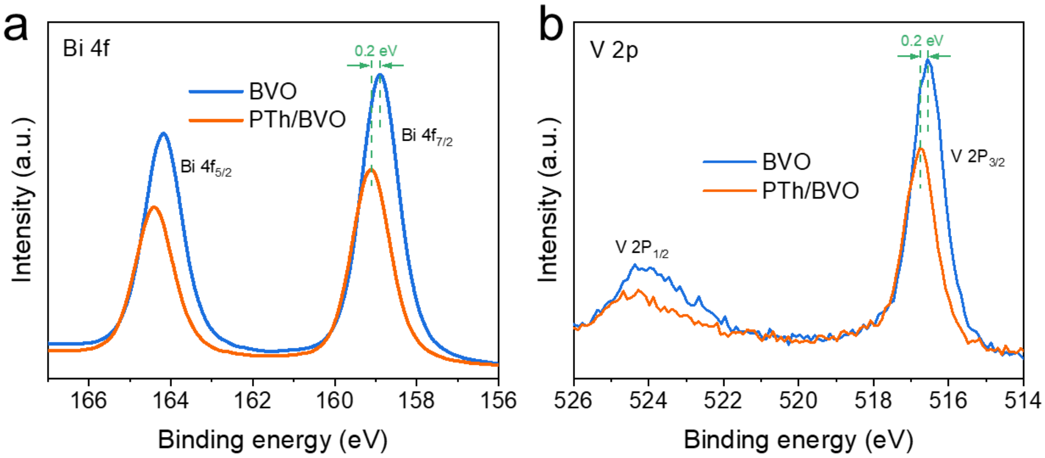


Supplementary Fig. 4. XPS spectra of a) Bi 4f, b) V 2p of the PTh/BVO and BVO.

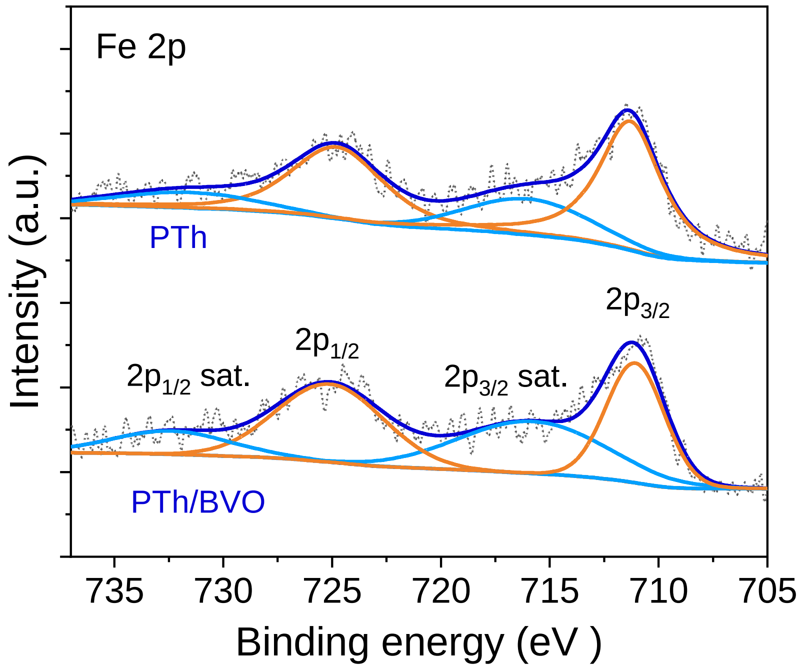


Supplementary Fig. 5. Fe 2p XPS spectra of BVO and PTh/BVO.

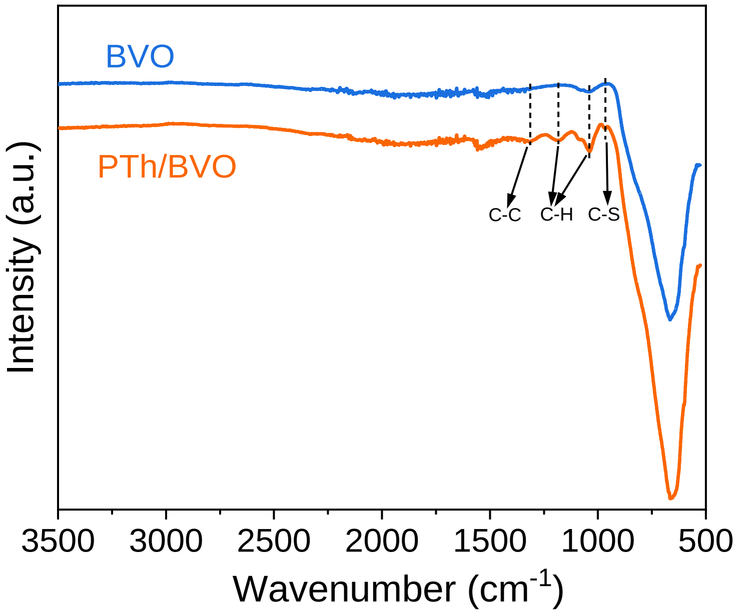


Supplementary Fig. 6. FTIR spectra of BVO and PTh/BVO.

The FTIR peak located at 968cm$^{-1}$ is the characteristic peak of C—S bonds.[1] The peaks located at 1044cm$^{-1}$ and 1182cm$^{-1}$ belong to C—H bond deformation of PTh, and the peak at 1315cm$^{-1}$ is the characteristic peak of C—C bonds of PTh, indicating that PTh layer is successfully coated on BVO photoanode.[2-4]

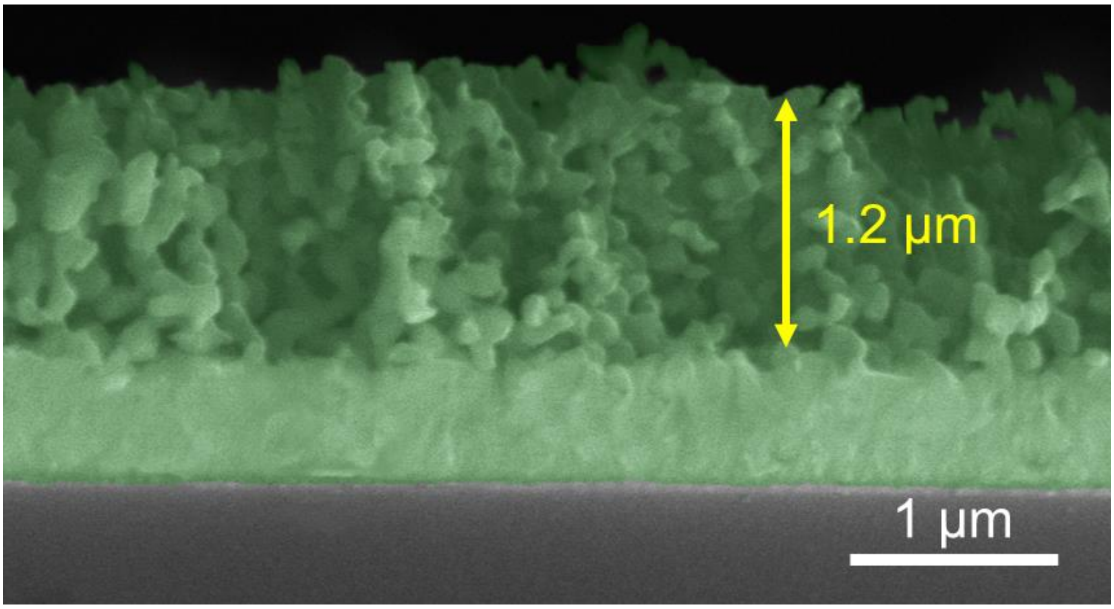


Supplementary Fig. 7. Cross-sectional SEM image of BVO.

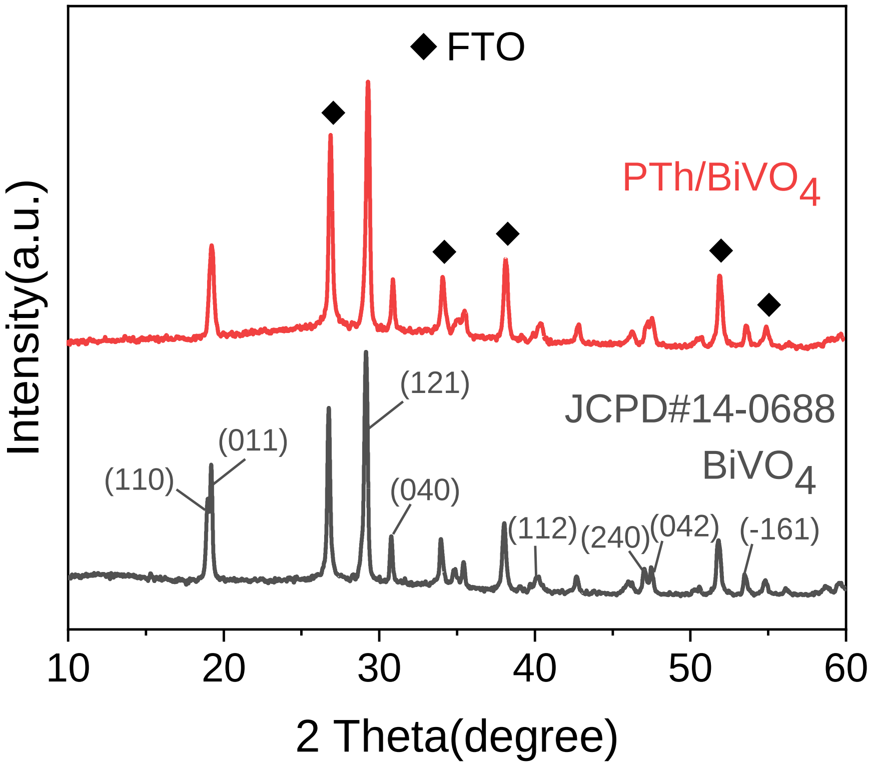


Supplementary Fig. 8. XRD patterns of PTh/BVO and BVO.

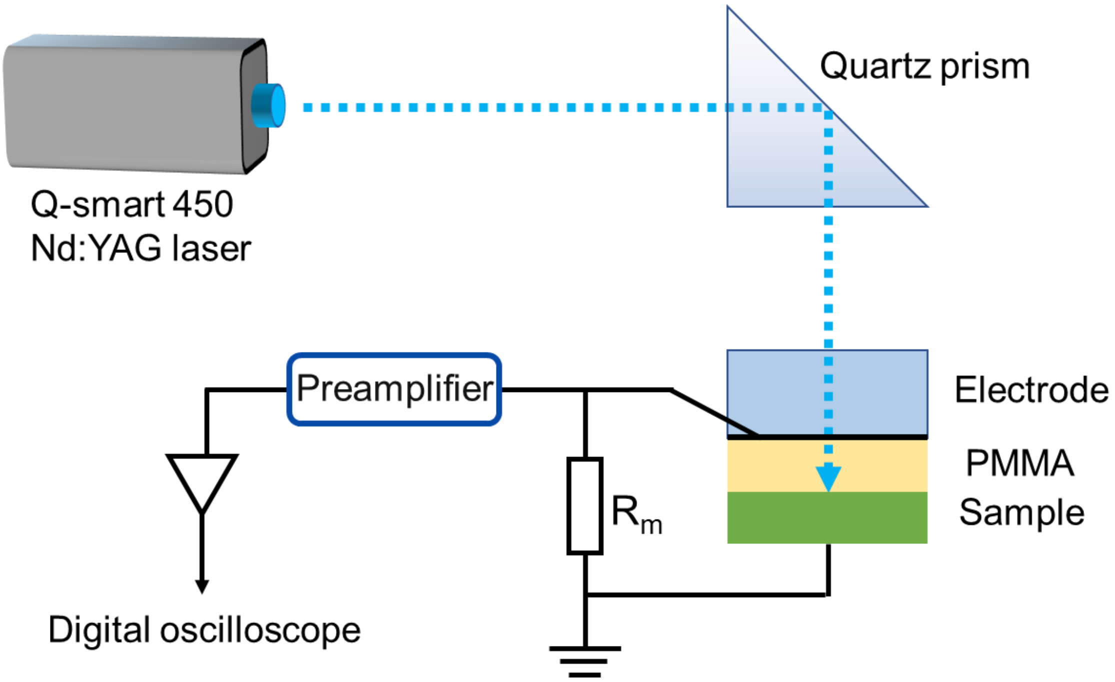


Supplementary Fig. 9. Schematic of TPV measurements.

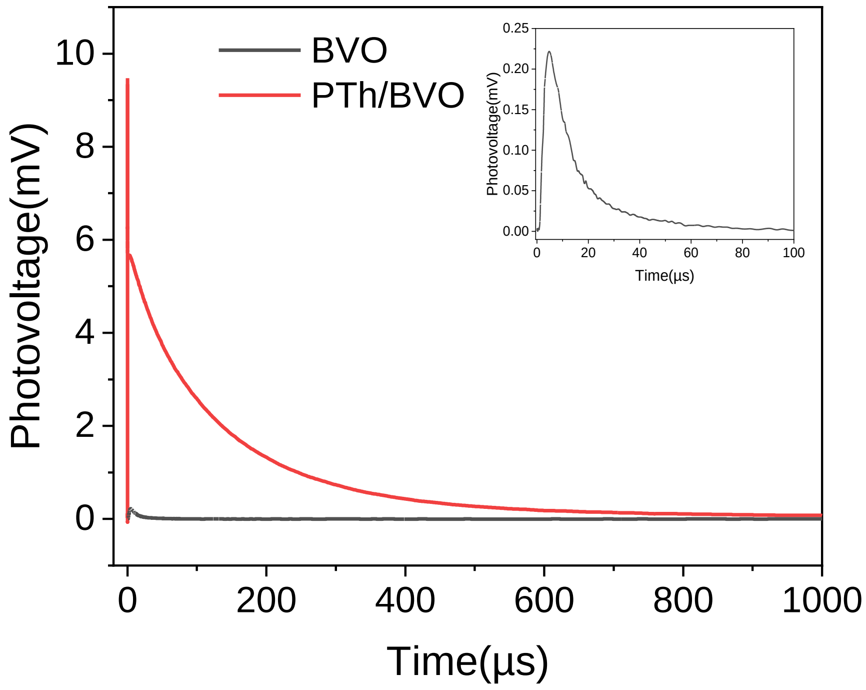


Supplementary Fig. 10. TS-SPV spectra of BVO and PTh/BVO on a normal time scale.

Inset: enlarged view of the weak signals of BVO.

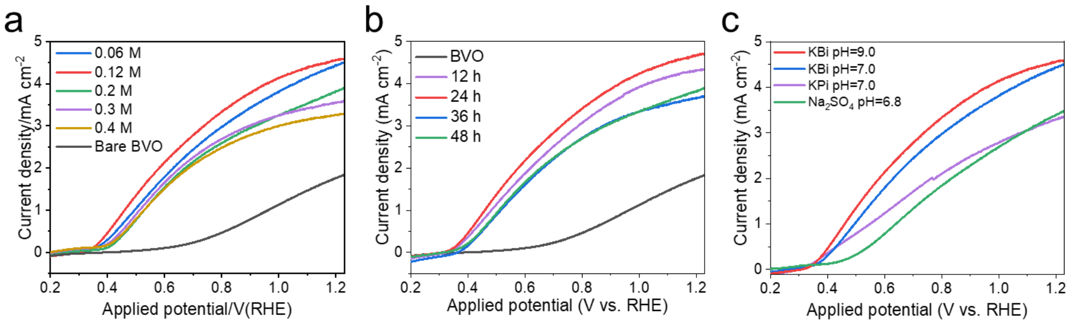


Supplementary Fig. 11. LSV curves of bare BVO and PTh/BVO with a) different concentrations of $FeCl_3$, b) different polymerization times, and c) various electrolytes.

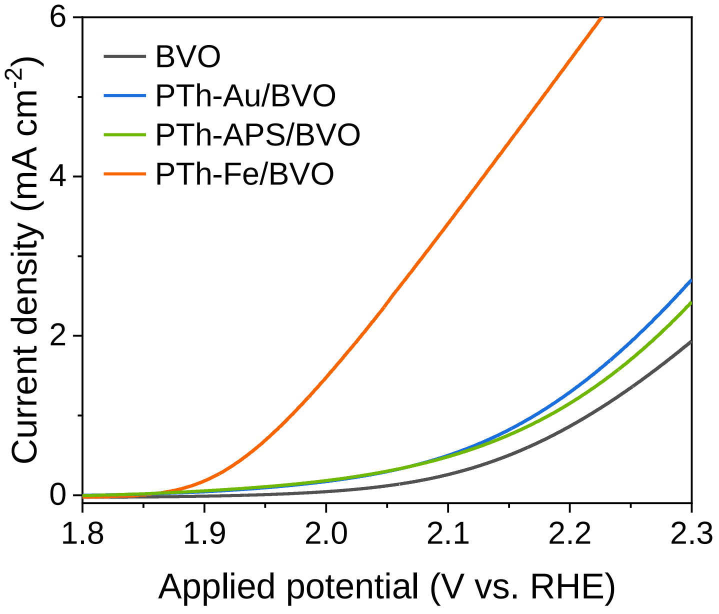


Supplementary Fig. 12. LSV curves of samples prepared by different initiators in dark.

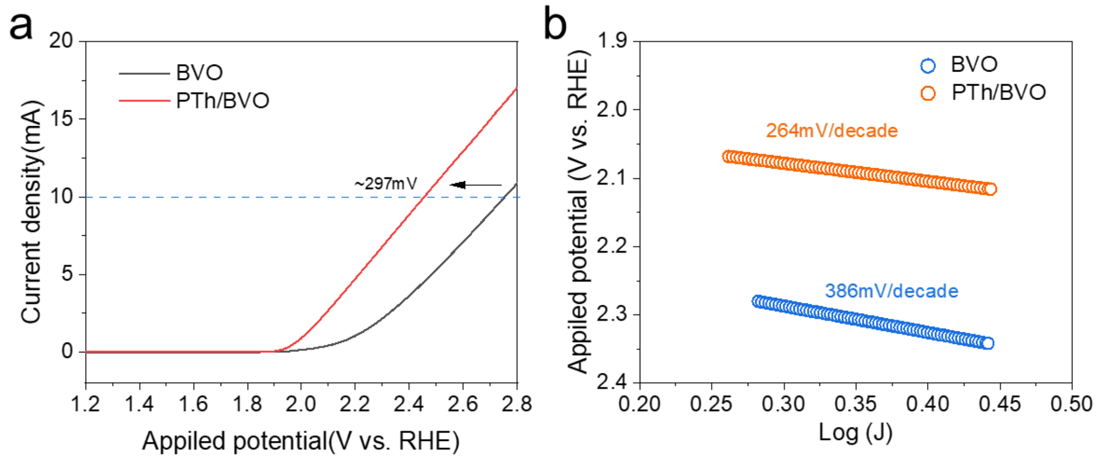


Supplementary Fig. 13. a) LSV curves in the dark; b) Tafel slope curves of BVO and PTh/BVO

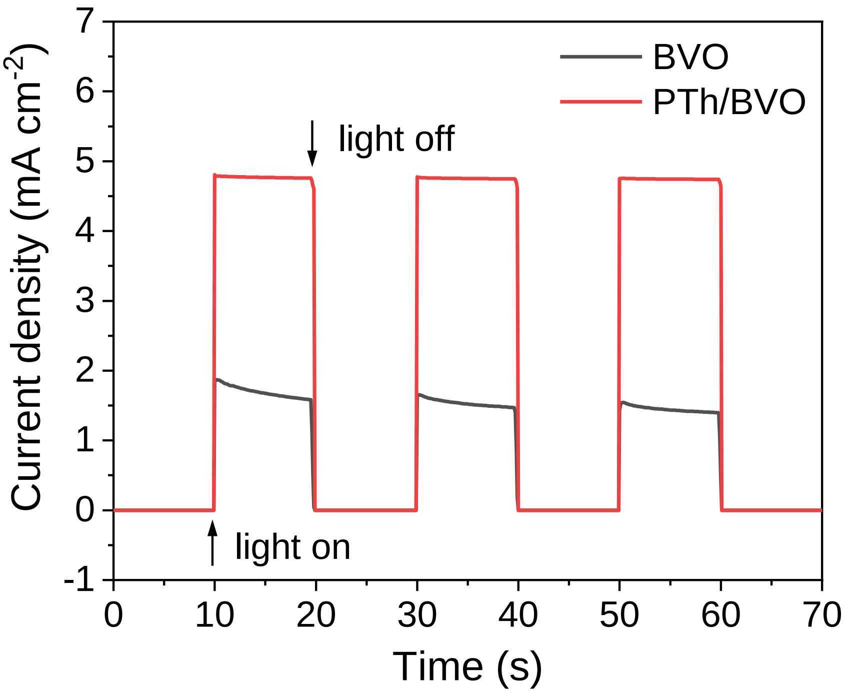


Supplementary Fig. 14. Chopped transient photocurrent density versus time of BVO and PTh/BVO at 1.23V vs. RHE.

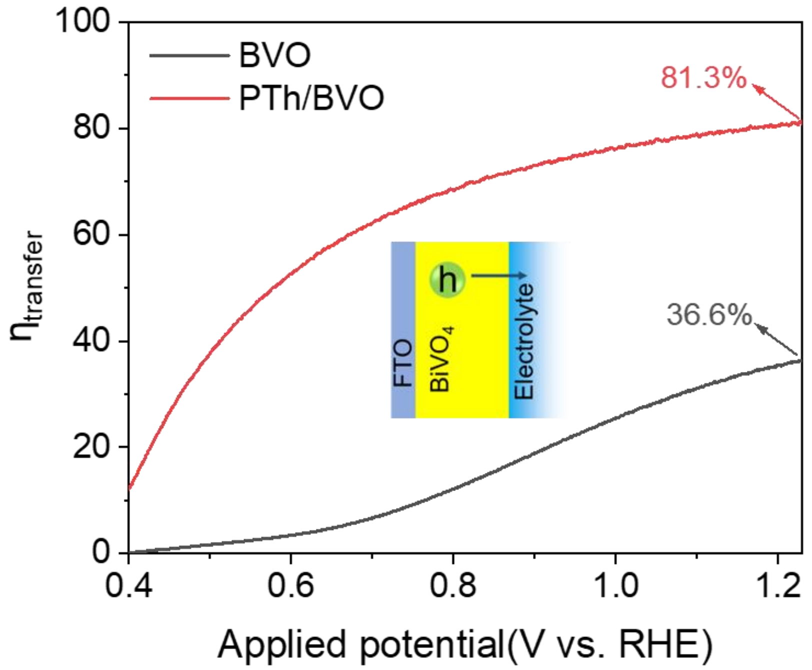


Supplementary Fig. 15. Charge transfer efficiencies of BVO and PTh/BVO.

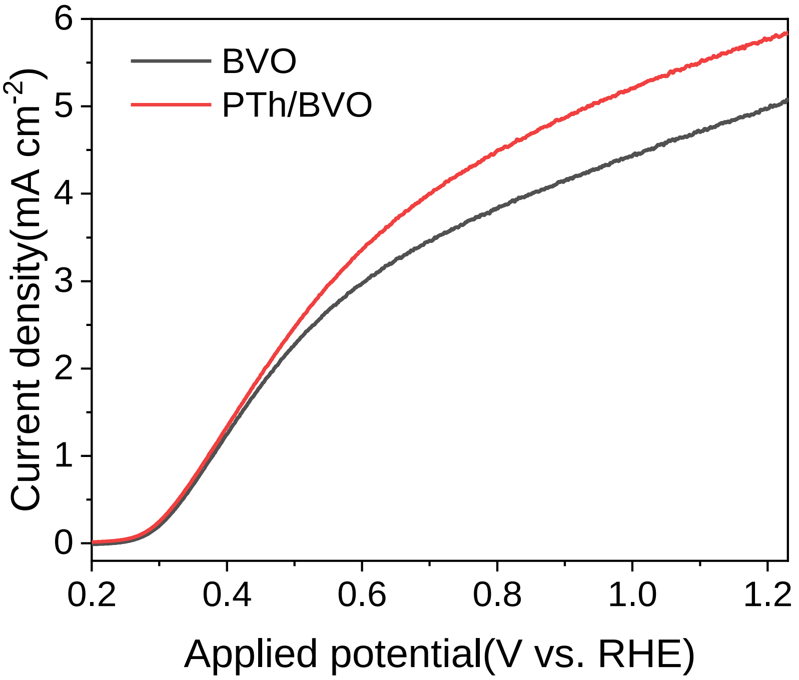


Supplementary Fig. 16. LSV curves with 0.1 M $Na_2SO_3$ as a hole scavenger in 0.5 M borate buffer (pH = 9.0) electrolyte.

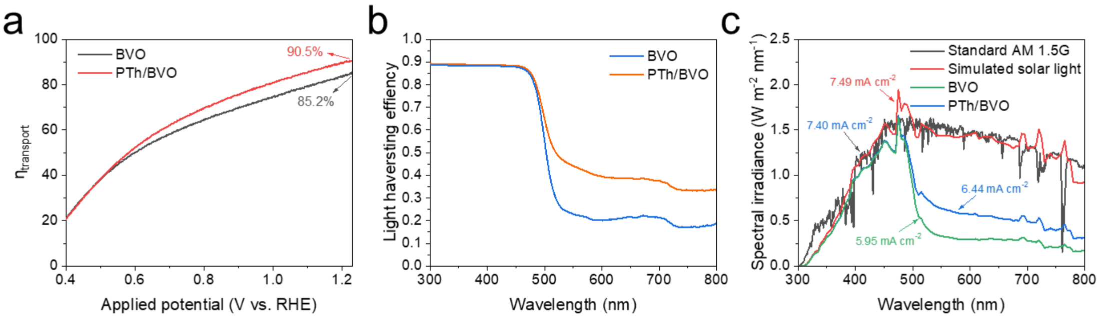


Supplementary Fig. 17. a) Charge separation efficiencies ($\eta_{transport}$), b) light harvesting efficiency (LHE), c) Spectra of the solar irradiance of AM 1.5G and corresponding $J_{abs}$ that are calculated by the LHE spectra of the BVO and PTh/BVO photoanodes

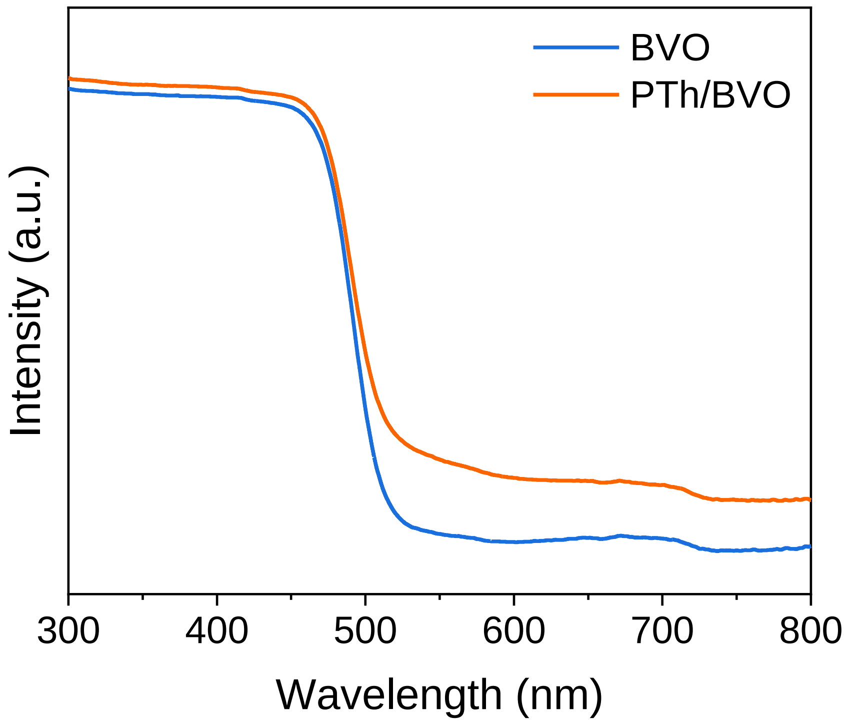


Supplementary Fig. 18. UV-vis spectra of BVO and PTh/BVO.

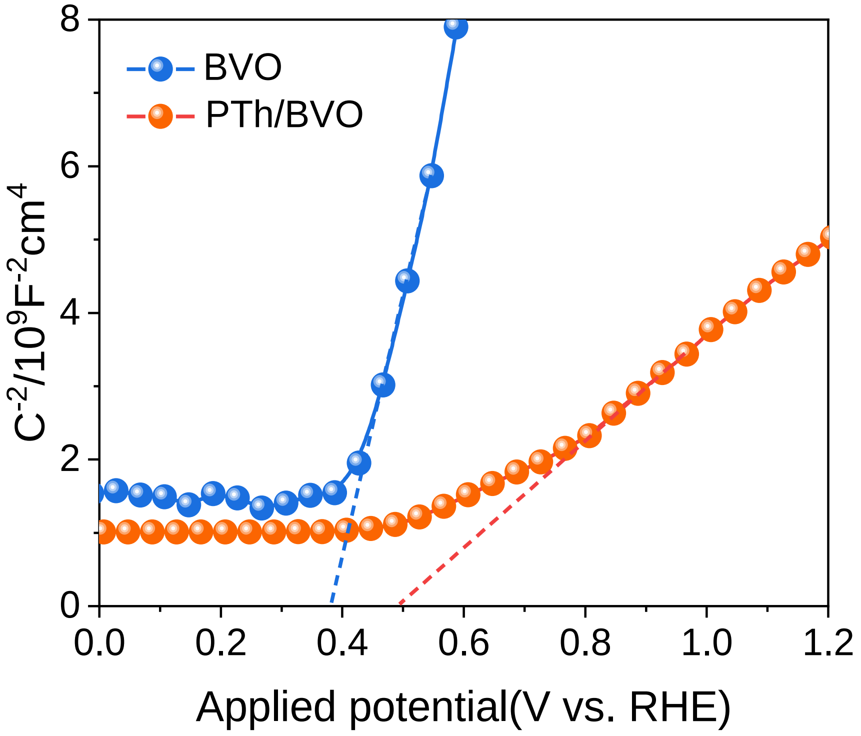


Supplementary Fig. 19. Mott–Schottky plots of BVO and PTh/BVO.

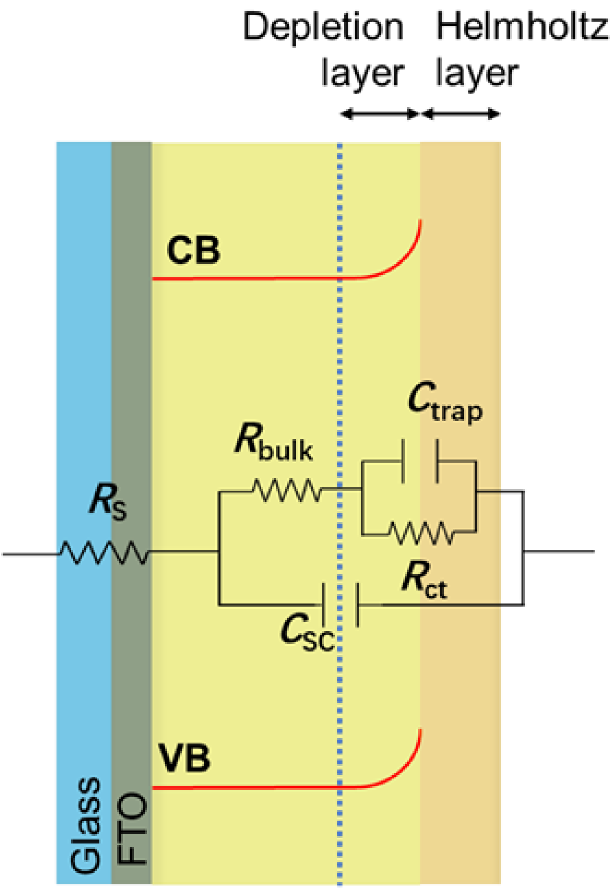


Supplementary Fig. 20. Equivalent circuit for the fitting and simulation of the EIS results.

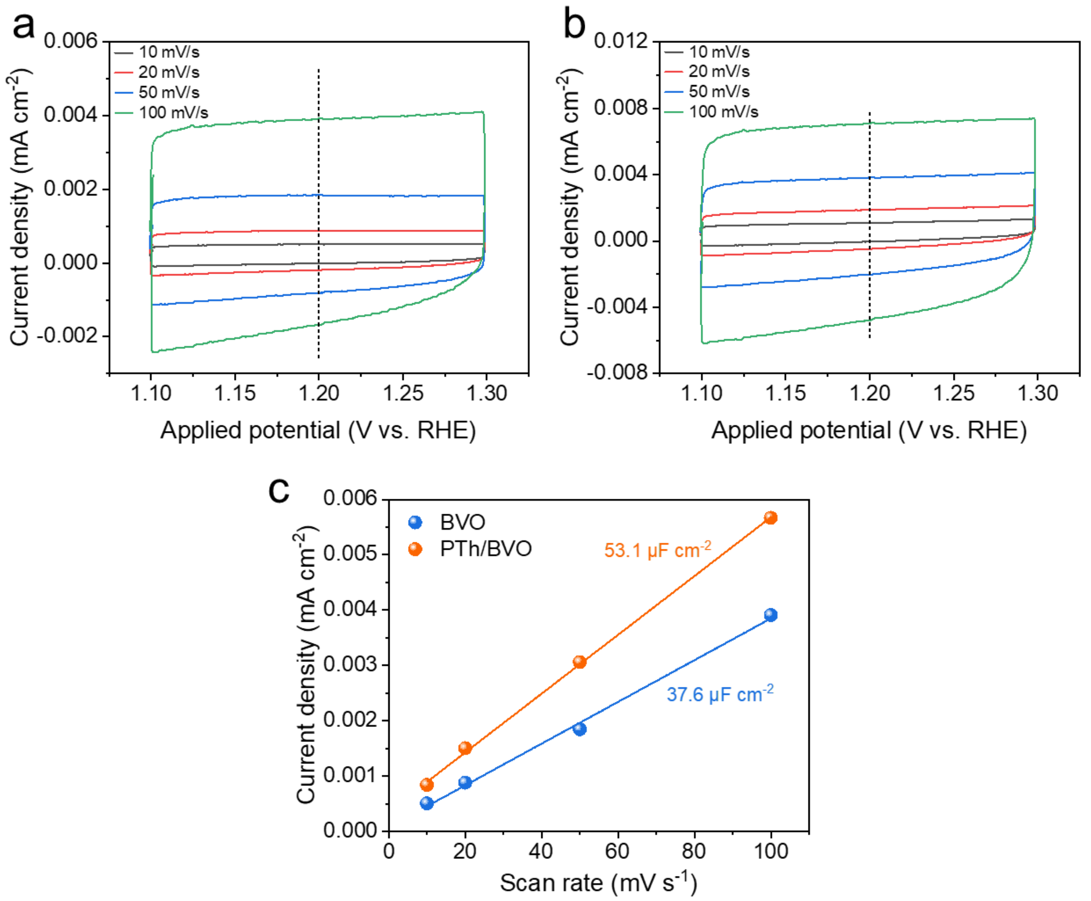


Supplementary Fig. 21. CV curves of a) BVO and b) PTh/BVO at various scanning rates. c) Capacitive current versus scan rate of BVO and PTh/BVO.

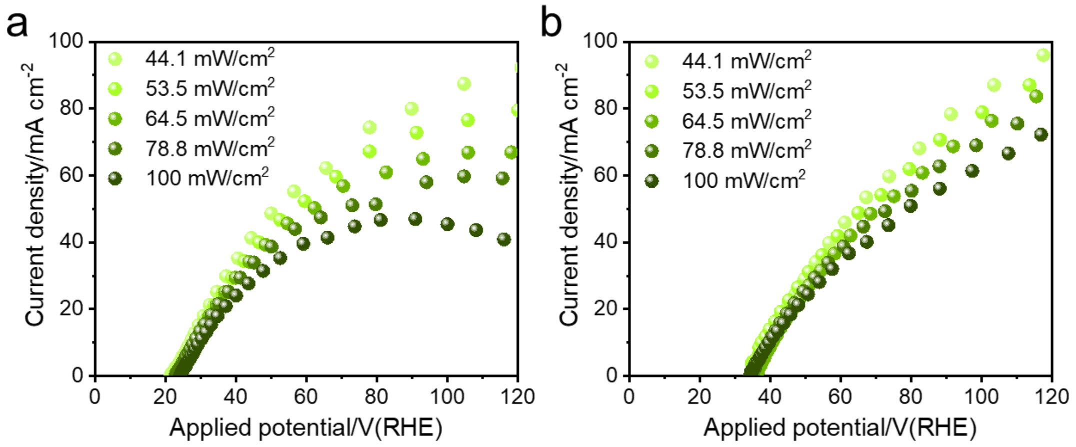


Supplementary Fig. 22. EIS data of a) PTh/BVO and b) BVO photoanodes. The impedance data were measured at the bias of 0.6 V vs. RHE.

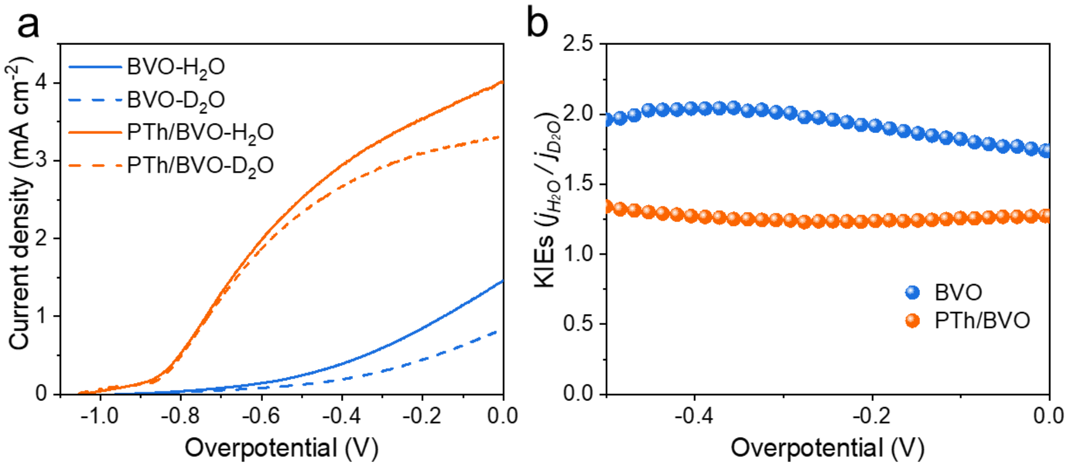


Supplementary Fig. 23. Figure 5. a) LSV curves of BVO and PTh/BVO in 0.1 M borate $H_2O$ and $D_2O$ solutions. b) KIE values of BVO and PTh/BVO.

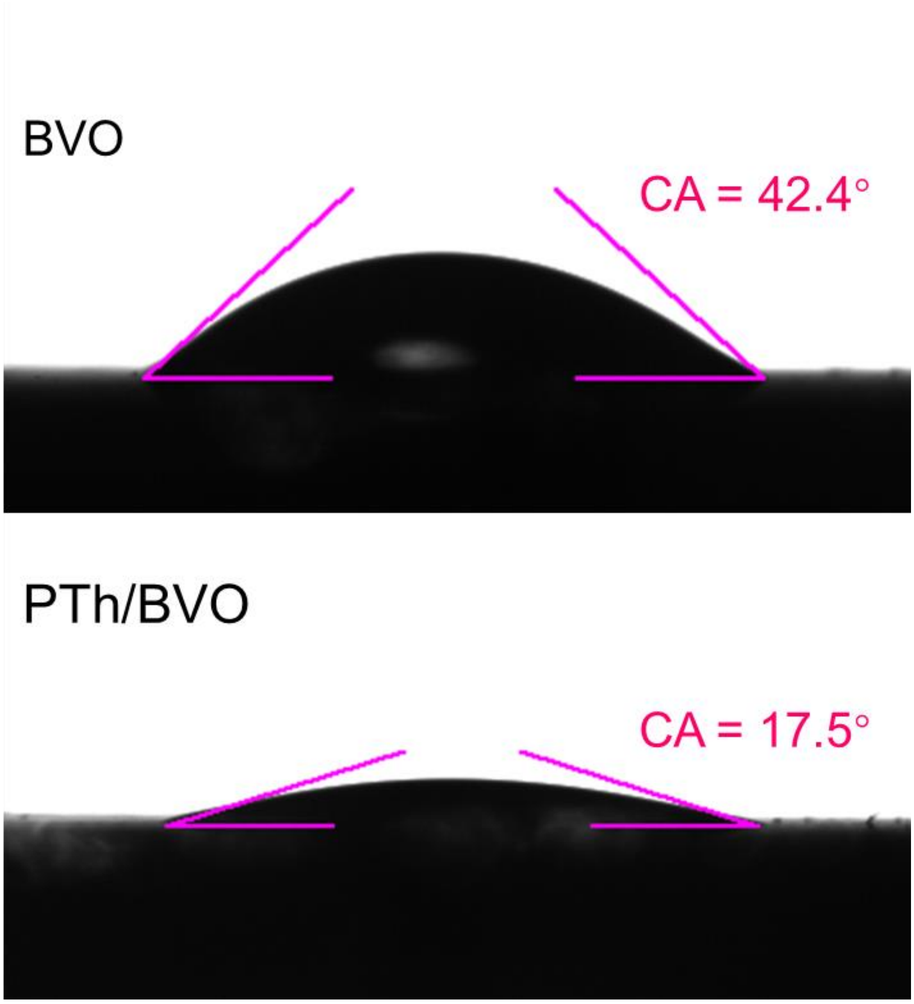

Supplementary Fig. 24. Contact angle of BVO and PTh/BVO.

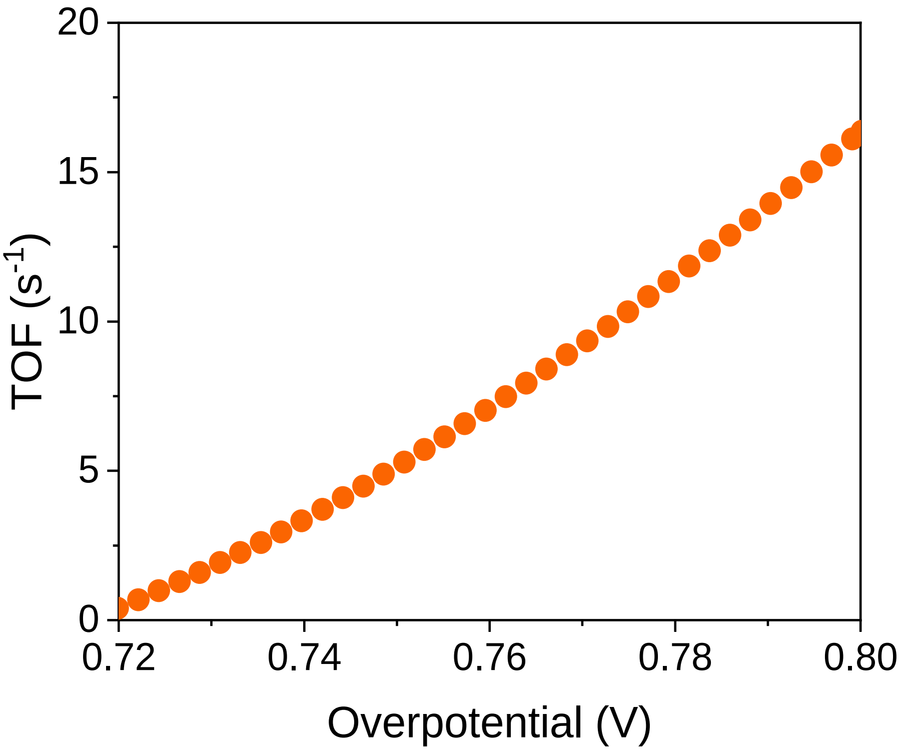


Supplementary Fig. 25. TOFs of PTh/BVO versus overpotential.

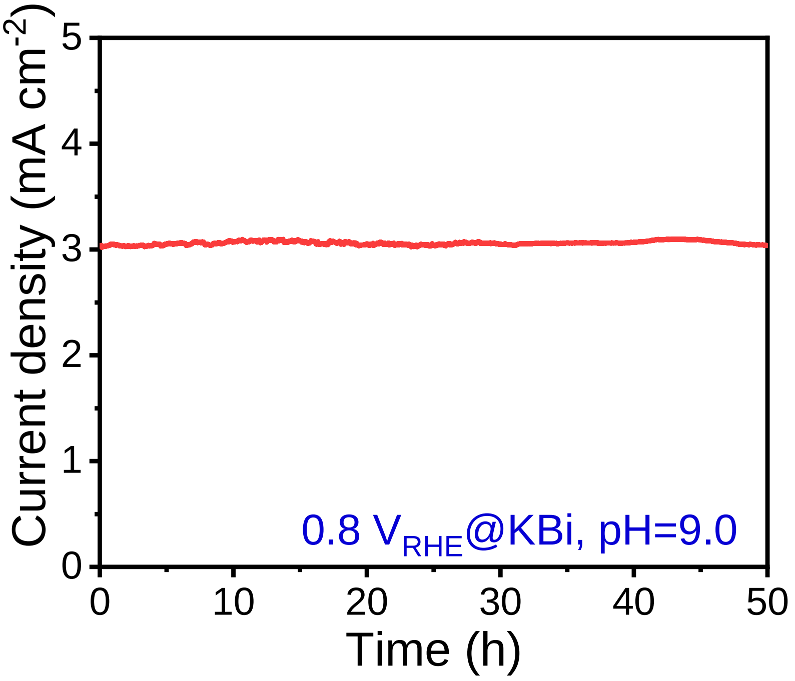


Supplementary Fig. 26. Long-term PEC stability of PTh/BVO at 0.8 V vs. RHE in 0.5 M electrolyte (pH = 9) under AM 1.5G illumination.

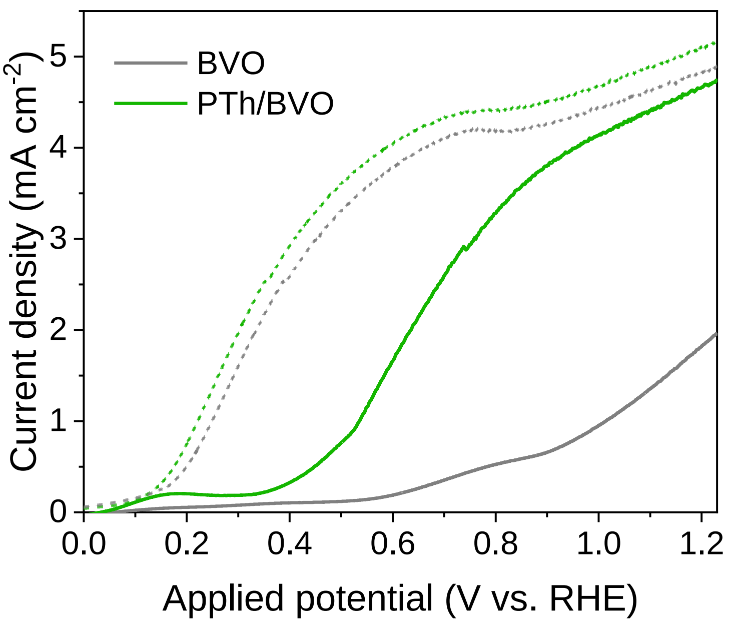


Supplementary Fig. 27. LSV curves of BVO and PTh/BVO in natural seawater under AM 1.5G illumination.

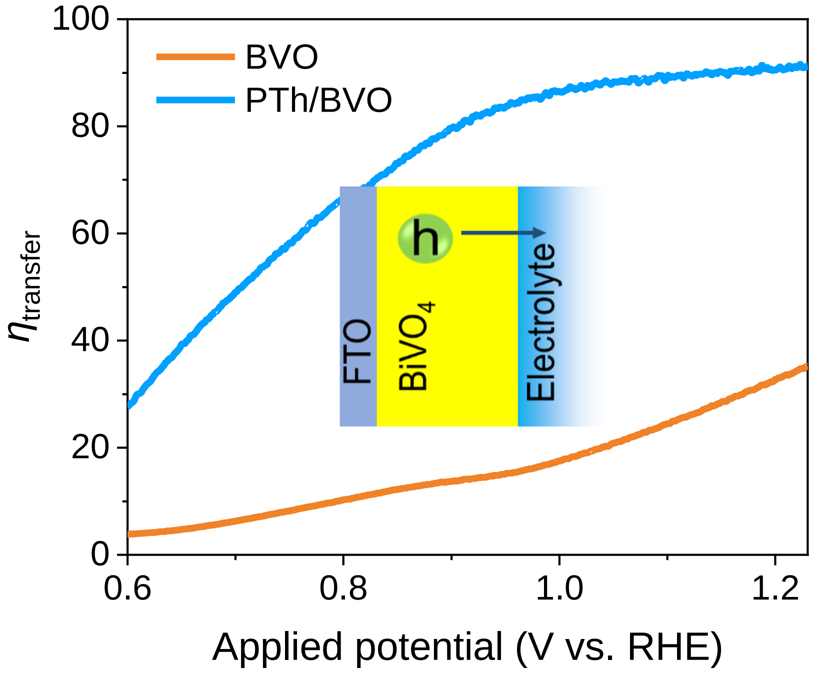


Supplementary Fig. 28. Charge transfer efficiency of PTh/BVO and BVO in PEC seawater splitting.

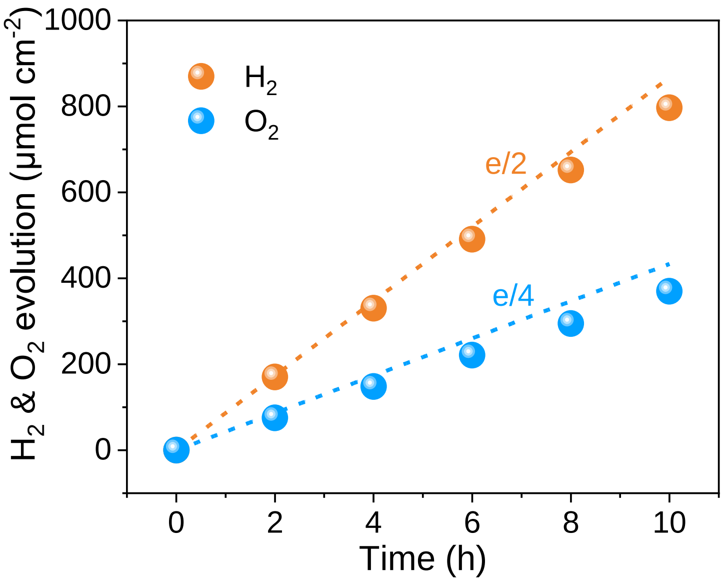


Supplementary Fig. 29. Time course of $O_2$ and $H_2$ gas evolution of PEC seawater splitting using PTh/BVO photoanode.

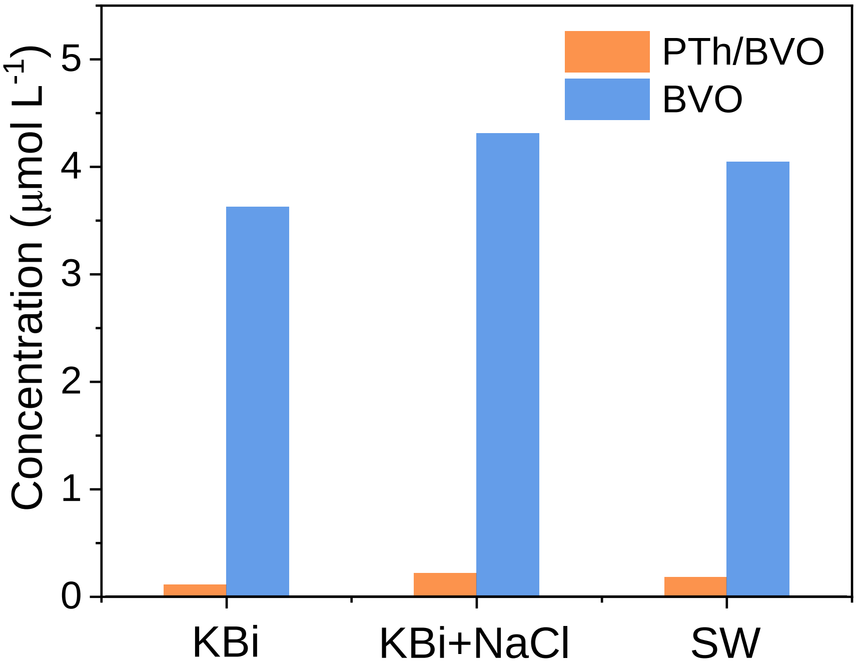


Supplementary Fig. 30. ICP-MS measurements for V element under i-t measurement for 40h.

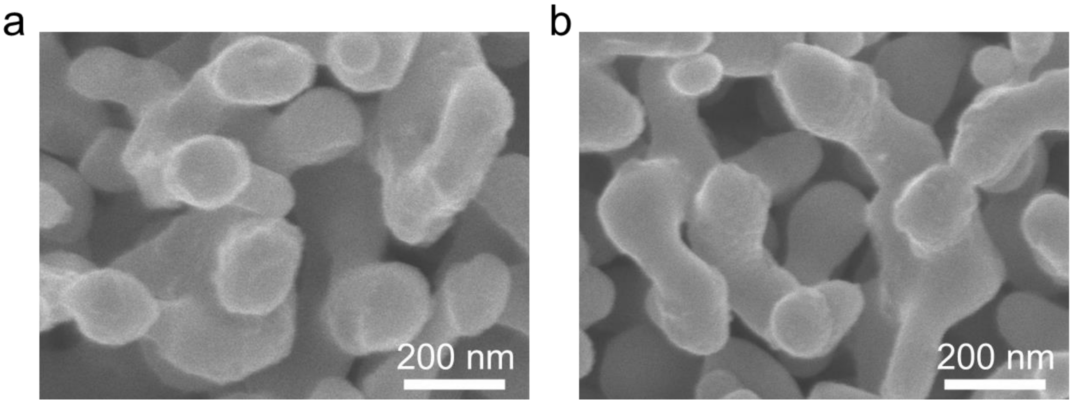


Supplementary Fig. 31. SEM images for PTh/BVO a) before i-t measurement and b) after 40h in seawater electrolyte.

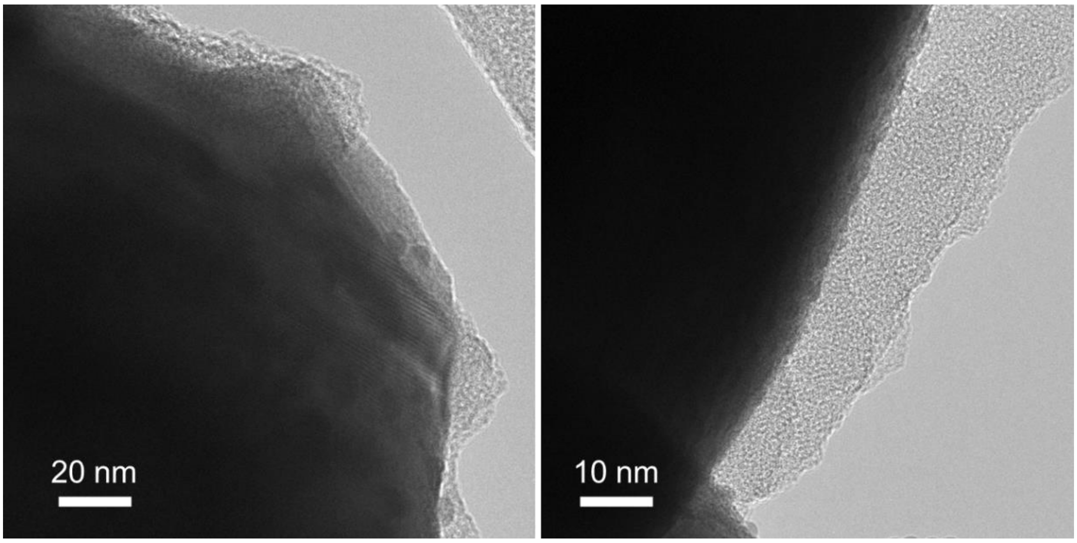


Supplementary Fig. 32. TEM images for PTh/BVO after i-t test in seawater electrolyte.

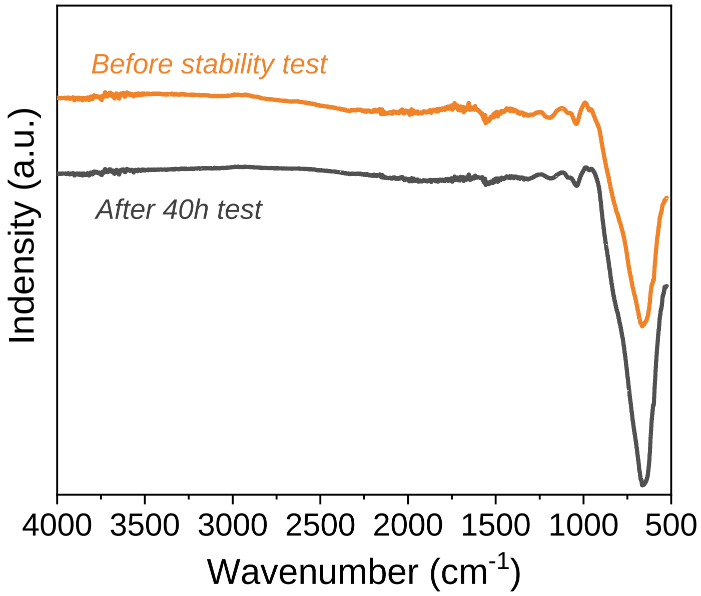


Supplementary Fig. 33. FTIR spectra of PTh/BVO.

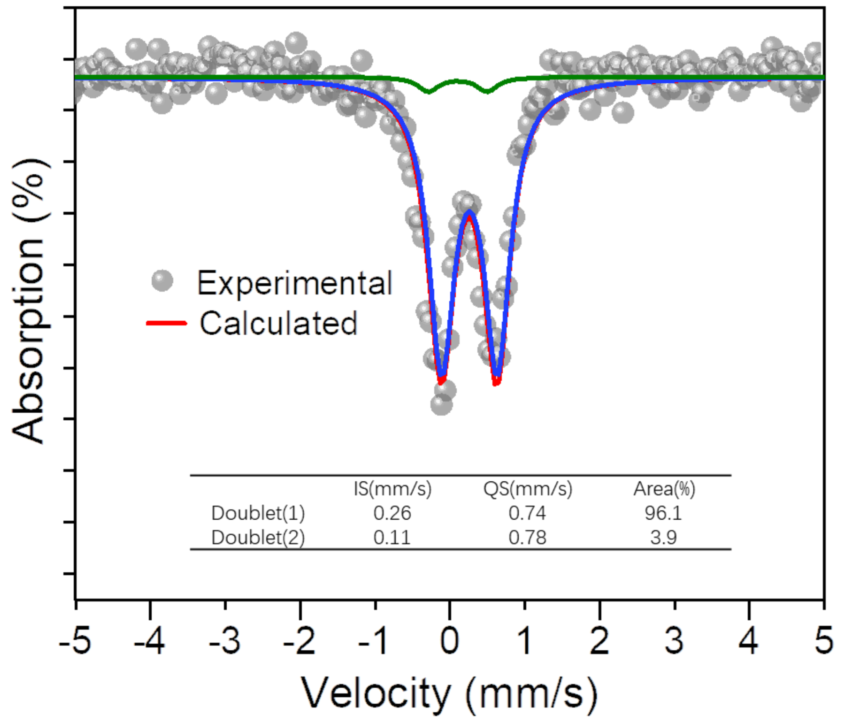


Supplementary Fig. 34. $^{57}$Fe Mössbauer spectra of PTh/BVO after i-t test.

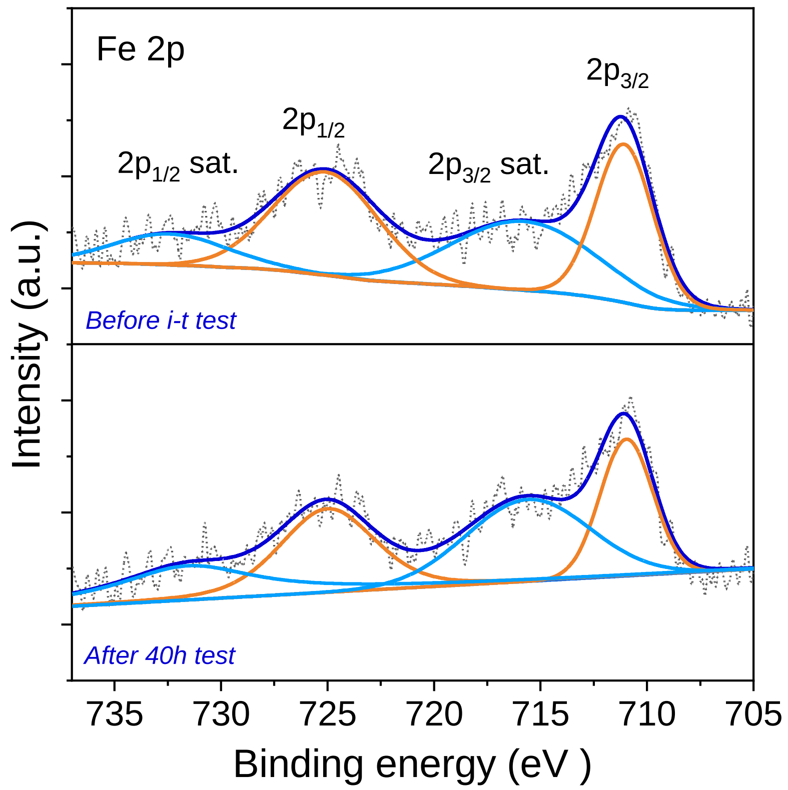


Supplementary Fig. 35. XPS Fe 2p spectra for PTh/BVO before i-t measurement and after 40h in seawater electrolyte.

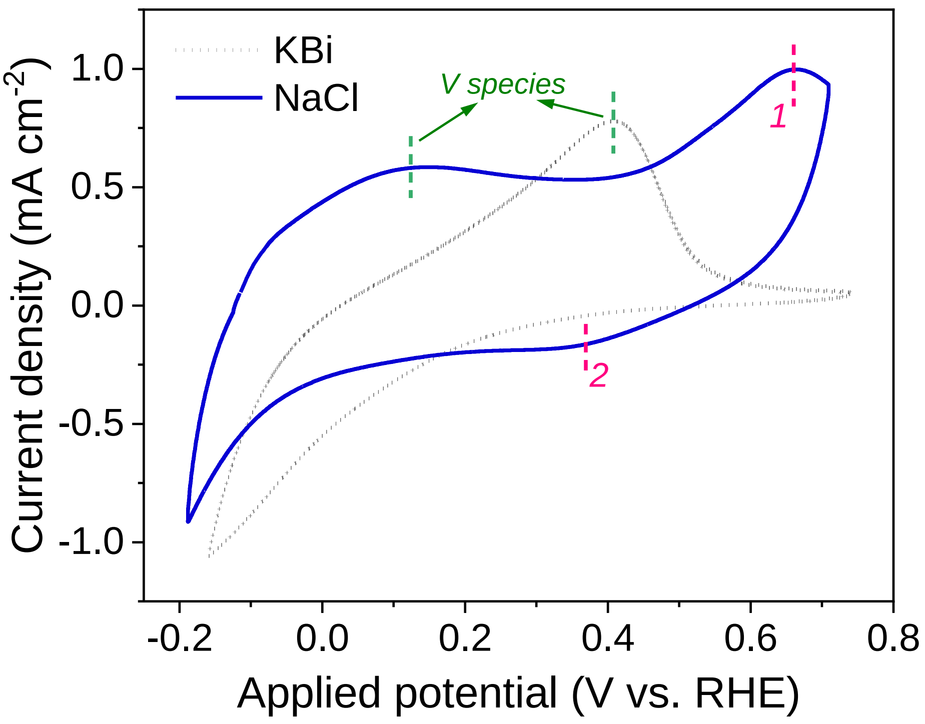


Supplementary Fig. 36. CV curves of PTh/BVO in 0.5 M KBi and 0.5 M NaCl electrolyte. Scan rate 20mV/s

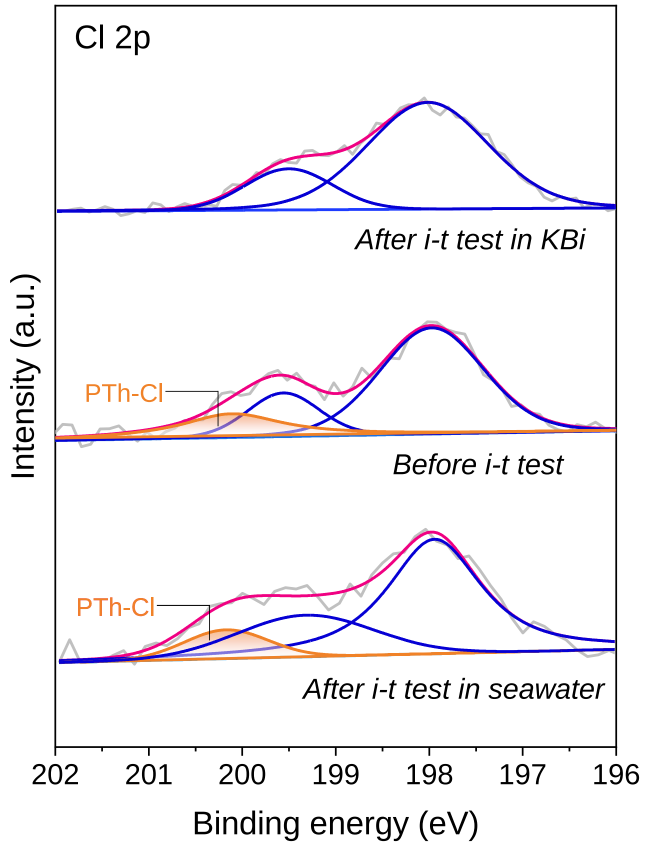


Supplementary Fig. 37. Cl 2p XPS spectra of PTh/BVO before and after i-t testing in KBi and seawater.

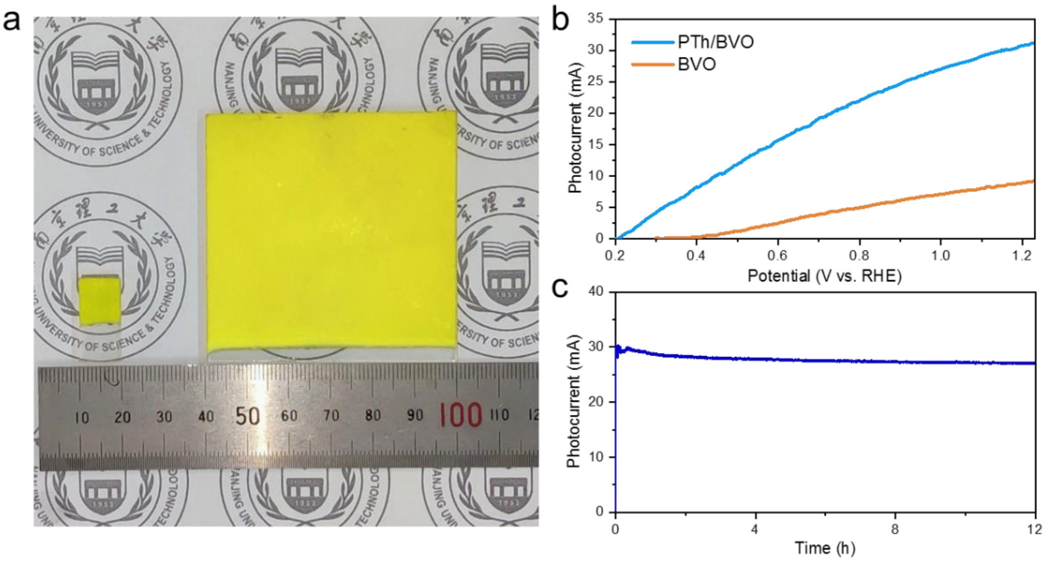


Supplementary Fig. 38. (a) Digital photographs of PTh/BVO photoanodes with different areas. (b) PEC performance of PTh/BVO and BVO with 30 $cm^2$. (c) Stability testing of PTh/BVO at 1.23 V vs. RHE in seawater electrolyte (pH = 8.1) under AM 1.5G illumination.

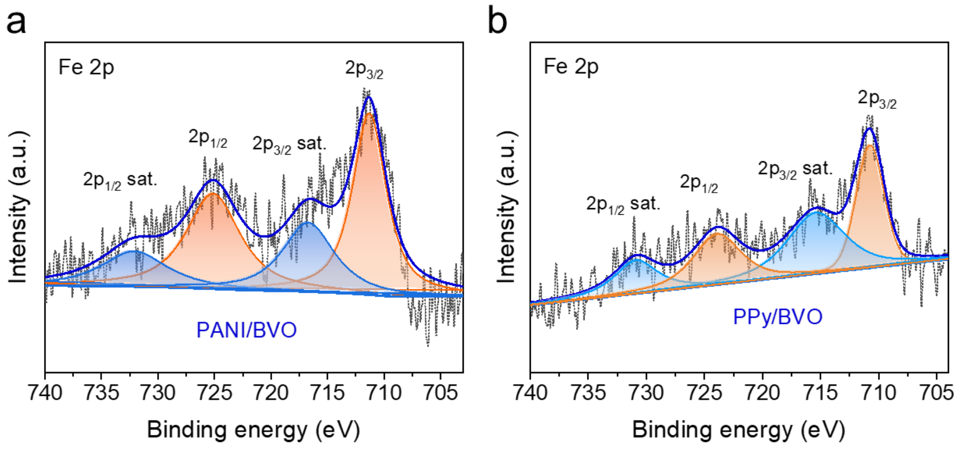


Supplementary Fig. 39. XPS spectra of Fe 2p of PANI/BVO and PPy/BVO.

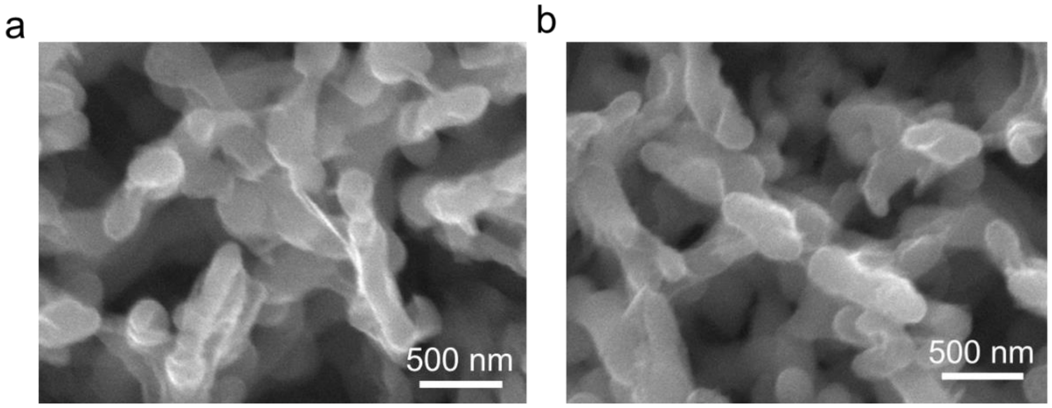


Supplementary Fig. 40. SEM image of a) PANI/BVO and b) PPy/BVO.

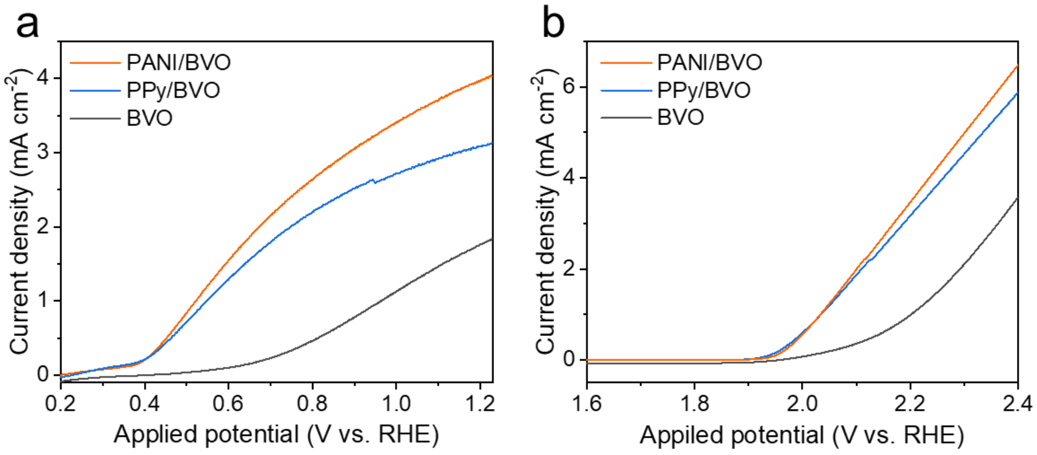


Supplementary Fig. 41. a) PEC performance and b) LSV curves in the dark of samples.

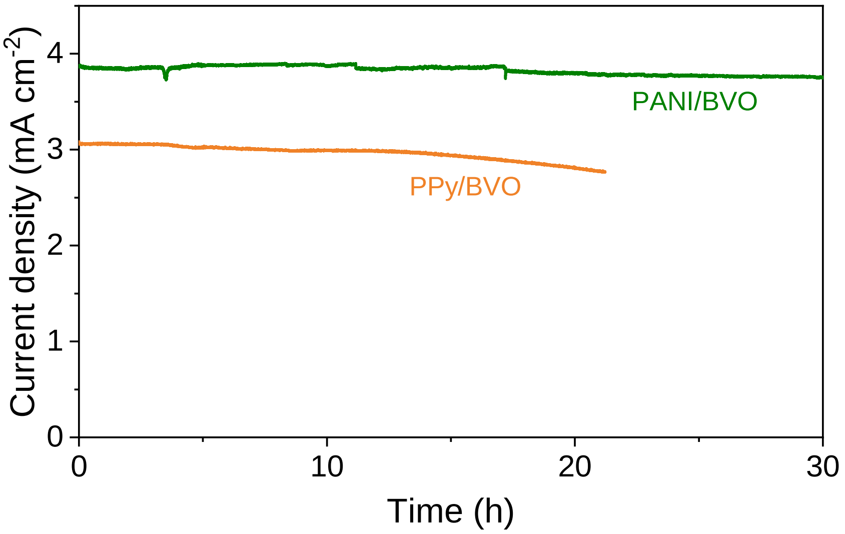


Supplementary Fig. 42. Long-term PEC stability of PPy/BVO and PANI/BVO at 1.23 V vs. RHE in seawater electrolyte (pH = 8.1) under AM 1.5G illumination.

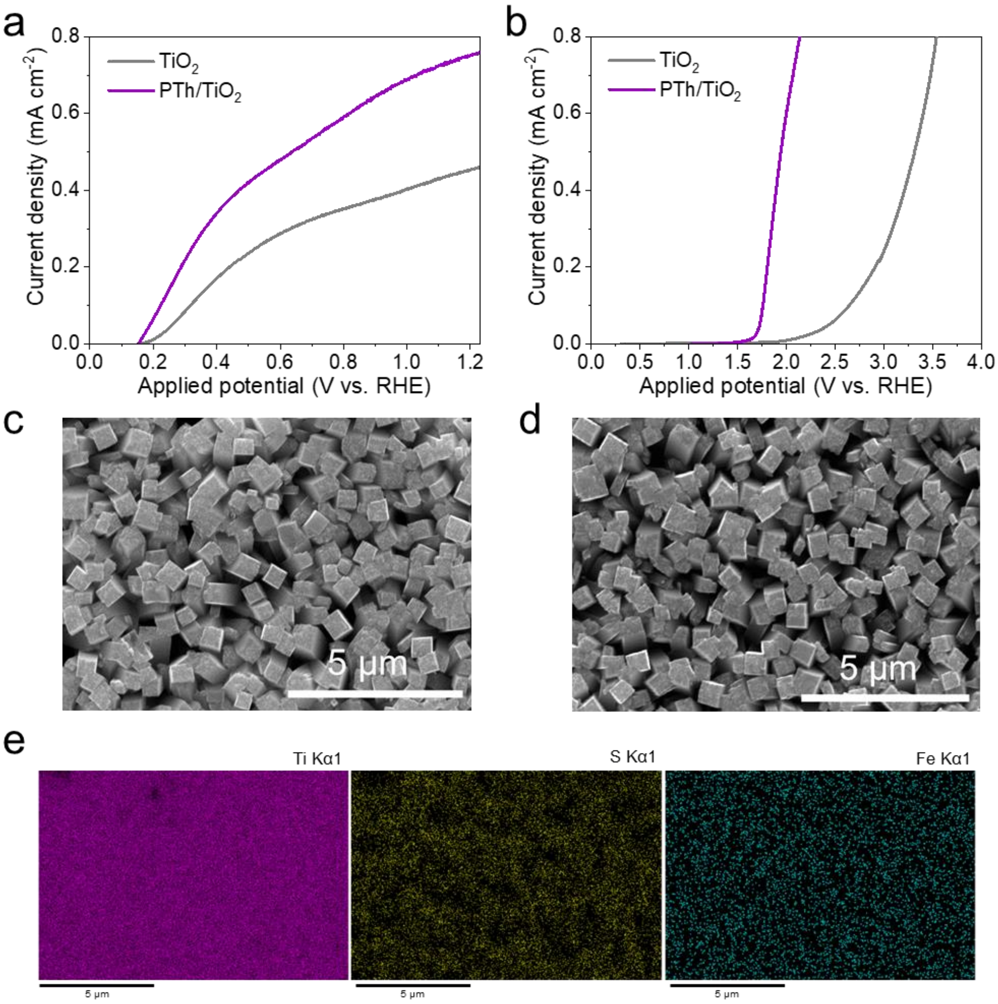


Supplementary Fig. 43. LSV curves of PTh/$TiO_2$ and $TiO_2$ photoanode in KBi (pH = 9) under (a) AM1.5G and (b) dark. SEM images of (c) $TiO_2$ and (d) PTh/$TiO_2$. (e) Corresponding EDS element maps.

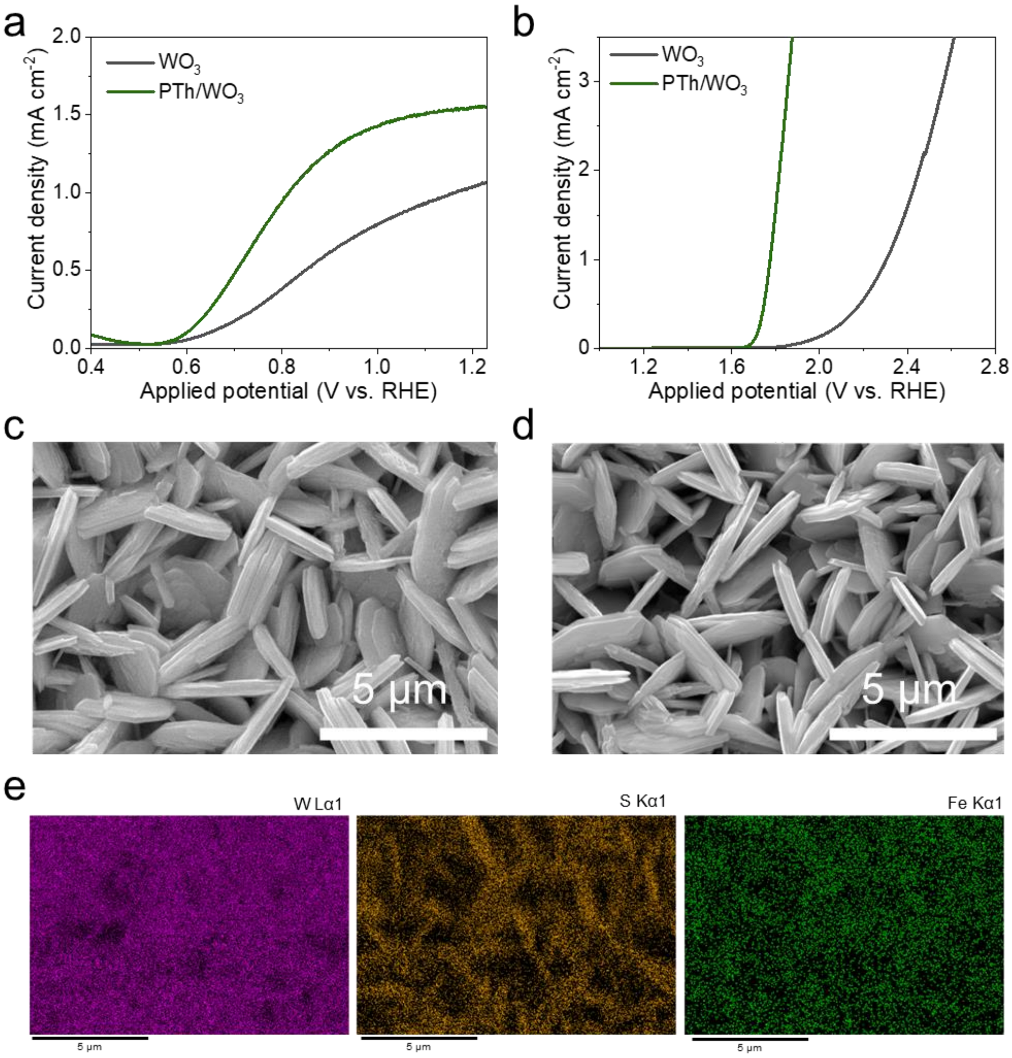


Supplementary Fig. 44. LSV curves of PTh/$WO_3$ and $WO_3$ photoanode in KBi (pH = 9) under (a) AM1.5G and (b) dark. SEM images of (c) $WO_3$ and (d) PTh/$WO_3$. (e) Corresponding EDS element maps.

**Supplementary Table 1.** Comparison of the PEC performance for the hybrid $BiVO_4$ photoanodes.

| Sample | Electrolyte (pH) | Current density (mA $cm^{-2}$) | Ref. |
|---|---|---|---|
| $BiVO_4$/CoFe-PA | 0.5 M NaBi (8.5) | 4.5 | 5 |
| $BiVO_4$@TANF | 0.5 M borate buffer (8.5) | 4.28 | 6 |
| ALD-$SnO_2$/$BiVO_4$/ NiFe-phenolic | 1.0 M NaBi (9.3) | 4.14 | 7 |
| $BiVO_4$/TACo | 0.15 M NaBi (8.5) | 4.8 | 8 |
| $BiVO_4$/TAFe | 0.15 M NaBi (9) | 3.7 | 9 |
| $BiVO_4$/PANI/Co-Pi | 0.5 M KPi (7) | 2.8 | 10 |
| CoNi-MOFs/$BiVO_4$ | 0.5 M $Na_2SO_4$ (6.8) | 3.2 | 11 |
| Co@CB[5]/$BiVO_4$ | 1.0 M borate buffer (9.2) | 4.8 | 12 |
| Co-salophen@Nafion/ $BiVO_4$ | 0.1 M KPi (7) | 4.27 | 13 |
| $[(cy)Ru(L_2bpy)OH_2]^+$/$BiVO_4$ | 0.1 M KPi (7.1) | 1.3 | 14 |
| CoTCPP/$Al_2O_3$/$BiVO_4$ | 0.1 M $Na_2SO_4$ (6.8) | 2.1 | 15 |
| PTh/$BiVO_4$ | 0.5 M borate buffer (9.0) | 4.72 | This work |

**Supplementary Table 2.** The pH and main components of the seawater in this work.

| ***Seawater*** *(from Tsingtao, China; pH=8.1)* | |
|---|---|
| ***Cations*** | ***Concentration* (mg/L)** |
| Na | 9731 |
| Mg | 993 |
| K | 297 |
| Ca | 332 |
| Sr | 4.48 |

**Supplementary Table 3.** Comparison of the Faradic efficiency ($H_2$) in seawater electrolyte with different photoanode materials.

| Photoanode | Faradic efficiency ($H_2$) | Ref. |
|---|---|---|
| Ti-$Fe_2O_3$-$In_2O_3$/CoOOH | 90% | 16 |
| Ag/$WO_3$/ZnFe-LDH | near 100% | 17 |
| $SrNbO_2N$/Nb | 90% | 18 |
| $WO_3$ | 80% | 19 |
| Co:$BiVO_4$ | 92% | 20 |
| PTh/BVO | 91.7% | This work |

## REFERENCES


1. Ma, Y.; Xu, Y.; Ji, X.; Xie, M.; Jiang, D.; Yan, J.; Song, Z.; Xu, H.; Li, H., Construction of polythiophene/$Bi_4O_5I_2$ nanocomposites to promote photocatalytic degradation of bisphenol a. *J. Alloys Compd.* **823**, 153773 (2020).

2. Kulkarni, G.; Kandesar, P.; Velhal, N.; Phadtare, V.; Jatratkar, A.; Shinde, S. K.; Kim, D.-Y.; Puri, ., Exceptional electromagnetic interference shielding and microwave absorption properties of room temperature synthesized polythiophene thin films with double negative characteristics (DNG) in the Ku-band region. *Chem. Eng. J.* **355**, 196-207 (2019).

3. Chen, R.; Chen, S.; Zhou, Y.; Wei, Z.; Wang, H.; Zheng, Y.; Li, M.; Sun, K.; Li, Y., Unsubstituted Polythiophene Film Deposited via In-Situ Sequential Solution Polymerization for Chemo-/Electrochromism. *Macromolecules* **53**, 4247-4254 (2020).

4. Faisal, M.; Harraz, F. A.; Jalalah, M.; Alsaiari, M.; Al-Sayari, S. A.; Al-Assiri, M. S., Polythiophene doped ZnO nanostructures synthesized by modified sol-gel and oxidative polymerization for efficient photodegradation of methylene blue and gemifloxacin antibiotic. *Mater. Today Commun.* **24,** 101048 (2020).

5. Sun, H.; Hua, W.; Li, Y.; Wang, J.-G., Conformal coating of superhydrophilic metal-organic complex toward substantially improved photoelectrochemical water oxidation. *Chem. Eng. J.* **427,** 131004 (2022).

6. Shi, Y.; Yu, Y.; Yu, Y.; Huang, Y.; Zhao, B.; Zhang, B., Boosting Photoelectrochemical Water Oxidation Activity and Stability of Mo-Doped $BiVO_4$

through the Uniform Assembly Coating of NiFe–Phenolic Networks. *ACS Energy Lett.* **3,** 1648-1654 (2018).

7. Bera, S.; Lee, S. A.; Lee, W.-J.; Kim, J.-H.; Kim, C.; Kim, H. G.; Khan, H.; Jana, S.; Jang, H. W.; Kwon, S.-H., Hierarchical Nanoporous $BiVO_4$ Photoanodes with High Charge Separation and Transport Efficiency for Water Oxidation. *ACS Appl. Mater. Interfaces* **13,** 14291-14301 (2021).

8. Tian, T.; Dong, C.; Liang, X.; Yue, M.; Ding, Y., Enhanced photoelectrochemical water oxidation activity of $BiVO_4$ by coating of Co-phenolic networks as hole-transfer and co-catalyst. *J. Catal.* **377**, 684-691 (2019).

9. Cao, X.; Xu, C.; Liang, X.; Ma, J.; Yue, M.; Ding, Y., Rationally designed/assembled hybrid $BiVO_4$-based photoanode for enhanced photoelectrochemical performance. *Appl. Catal., B* **260**, 118136 (2020).

10. Zhao, M.; Chen, T.; He, B.; Hu, X.; Huang, J.; Yi, P.; Wang, Y.; Chen, Y.; Li, Z.; Liu, X., Photothermal effect-enhanced photoelectrochemical water splitting of a $BiVO_4$ photoanode modified with dual-functional polyaniline. *J. Mater. Chem. A* **8**, 15976-15983 (2020).

11. Zhou, S.; Chen, K.; Huang, J.; Wang, L.; Zhang, M.; Bai, B.; Liu, H.; Wang, Q., Preparation of heterometallic CoNi-MOFs-modified $BiVO_4$: a steady photoanode for improved performance in photoelectrochemical water splitting. *Appl. Catal., B* **266**, 118513 (2020).

12. Li, F.; Yang, H.; Zhuo, Q.; Zhou, D.; Wu, X.; Zhang, P.; Yao, Z.; Sun, L., A Cobalt@Cucurbit[5]uril Complex as a Highly Efficient Supramolecular Catalyst

for Electrochemical and Photoelectrochemical Water Splitting. *Angew. Chem. Int. Ed.* **60**, 1976-1985 (2021).

13. Liu, Y.; Jiang, Y.; Li, F.; Yu, F.; Jiang, W.; Xia, L., Molecular cobalt salophen catalyst-integrated $BiVO_4$ as stable and robust photoanodes for photoelectrochemical water splitting. *J. Mater. Chem. A* **6**, 10761-10768 (2018).

14. de Respinis, M.; Joya, K. S.; De Groot, H. J. M.; D'Souza, F.; Smith, W. A.; van de Krol, R.; Dam, B., Solar Water Splitting Combining a $BiVO_4$ Light Absorber with a Ru-Based Molecular Cocatalyst. *J. Phys. Chem. C* **119**, 7275-7281 (2015).

15. Liu, B.; Li, J.; Wu, H.-L.; Liu, W.-Q.; Jiang, X.; Li, Z.-J.; Chen, B.; Tung, C.-H.; Wu, L.-Z., Improved Photoelectrocatalytic Performance for Water Oxidation by Earth-Abundant Cobalt Molecular Porphyrin Complex-Integrated $BiVO_4$ Photoanode. *ACS Appl. Mater. Interfaces* **8**, 18577-18583 (2016).

16 Wang, S. *et al.* Boosted Photoelectrochemical Seawater Splitting by CoOOH-Modified Ti-$Fe_2O_3$/$In_2O_3$ by Synergy of Electronic Modulation and Heterojunction Construction. *ACS Appl. Nano Mater.* **6**, 20240-20250 (2023).

17 Liu, J., Xu, S.-M., Li, Y., Zhang, R. & Shao, M. Facet engineering of $WO_3$ arrays toward highly efficient and stable photoelectrochemical hydrogen generation from natural seawater. *Appl. Catal., B* **264**, 118540 (2020).

18 Trinh, V.-H. & Seo, J. Porous Cuboidal $SrNbO_2N$ Crystals Grown on a Nb Substrate as an Active Photoanode for Neutral Seawater Splitting under Sunlight. *ACS Sustainable Chem. Eng.* **11**, 1655-1665 (2023).

19 Jadwiszczak, M., Jakubow-Piotrowska, K., Kedzierzawski, P., Bienkowski, K. &

Augustynski, J. Highly Efficient Sunlight-Driven Seawater Splitting in a Photoelectrochemical Cell with Chlorine Evolved at Nanostructured $WO_3$ Photoanode and Hydrogen Stored as Hydride within Metallic Cathode. *Adv. Energy Mater.* **10**, 1903213 (2020).

20 Chauhan, I. *et al.* Nanostructured Co-doped $BiVO_4$ for efficient and sustainable photoelectrochemical chlorine evolution from simulated sea-water. *Dalton Trans.* **52**, 2051-2061 (2023).
